# Bipartite electronic superstructures in the vortex core of Bi_2_Sr_2_CaCu_2_O_8+*δ*_

**DOI:** 10.1038/ncomms11747

**Published:** 2016-05-27

**Authors:** T. Machida, Y. Kohsaka, K. Matsuoka, K. Iwaya, T. Hanaguri, T. Tamegai

**Affiliations:** 1 RIKEN Center for Emergent Matter Science, Wako, Saitama 351-0198, Japan; 2 Department of Applied Physics, The University of Tokyo, Hongo, Bunkyo-ku, Tokyo 113-8656, Japan

## Abstract

The central issue in the physics of cuprate superconductivity is the mutual relationship among superconductivity, pseudogap and broken-spatial-symmetry states. A magnetic field *B* suppresses superconductivity, providing an opportunity to investigate the competition among these states. Although various *B*-induced electronic superstructures have been reported, their energy, spatial and momentum-space structures are unclear. Here, we show using spectroscopic-imaging scanning tunnelling microscopy on Bi_2_Sr_2_CaCu_2_O_8+*δ*_ that there are two distinct *B*-induced electronic superstructures, both being localized in the vortex core but appearing at different energies. In the low-energy range where the nodal Bogoliubov quasiparticles are well-defined, we observe the so-called vortex checkerboard that we identify as the *B*-enhanced quasiparticle interference pattern. By contrast, in the high-energy region where the pseudogap develops, the broken-spatial-symmetry patterns that pre-exist at *B*=0 T is locally enhanced in the vortex core. This evidences the competition between superconductivity and the broken-spatial-symmetry state that is associated with the pseudogap.

## Abstract (web summary)

Field-induced electronic structures with spatial, momentum and energy resolution reveal the nature of interaction among multiple phases in correlated materials. Here, Machida *et al*. report two magnetic field-induced electronic superstructures in Bi_2_Se_2_CaCu_2_O_8+*δ*_, evidencing competition between superconductivity and emerging states.

## Graphical abstract

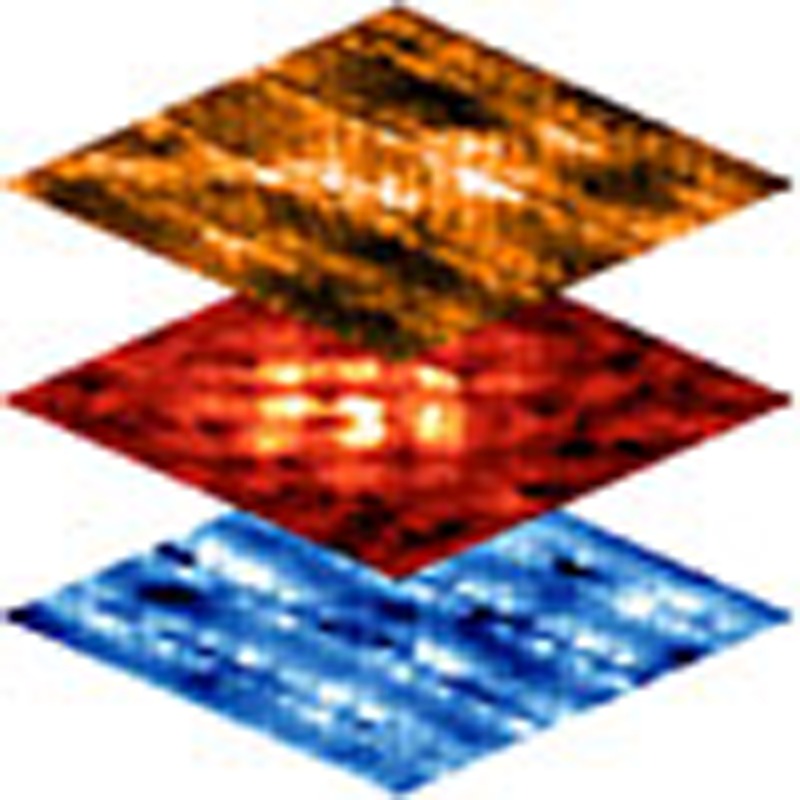

The electronic states in cuprates exhibit distinct features depending on energy and momentum^1^,^2^. The low-energy near-nodal states host the homogeneous *d*-wave superconductivity that manifests itself in the Bogoliubov quasiparticle interference (BQPI) patterns imaged by spectroscopic-imaging scanning tunnelling microscopy (SI-STM)^2^,^3^,^4^,^5^. BQPI is no longer observed above the doping-dependent extinction energy Δ_0_ and outside the diagonal line in momentum-space connecting (*π*/*a*_0_, 0) and (0, *π*/*a*_0_), where *a*_0_ denotes Cu–O–Cu distance^2^. The higher-energy states near the antinode are governed by the pseudogap whose apparent magnitude Δ_1_ is spatially inhomogeneous and the quasiparticle excitations near Δ_1_ break rotational and translational symmetries of the CuO_2_ plane^2^,^6^,^7^,^8^,^9^,^10^,^11^,^12^.

To establish the relationship among these electronic states, it is indispensable to investigate how the pseudogap and the broken-spatial-symmetry state are affected when the superconductivity is suppressed. The application of *B* is one of the ways to suppress superconductivity. It has been shown in La- and Y-based cuprates that the electronic orders that break the spatial symmetry are enhanced or even generated by *B*^13^,^14^,^15^,^16^,^17^,^18^,^19^. However, the detailed energy, spatial and momentum-space structures of the *B*-enhanced orders are unknown. To address this issue, we utilize SI-STM in *B* owing to the following three advantages. First, *B* can suppress superconductivity at the lowest temperatures where the thermal broadening effect is negligible, making it possible to study the precise energy scale of the phenomenon. Second, atomic-scale spatial resolution of SI-STM is highly beneficial not only to identify the locations of vortices but also to determine the real-space structure of vortex-induced states. Finally, by using the Fourier transformation, SI-STM acquires the momentum-space resolution even under *B* that enables us to discuss the near-nodal and antinodal states separately.

We choose optimally doped Bi_2_Sr_2_CaCu_2_O_8+*δ*_ (superconducting transition temperature *T*_*c*_∼90 K) as a sample because of its high-quality surface necessary for SI-STM. Pioneering SI-STM studies of the vortices in Bi-based cuprates have discovered that an electronic superstructure, so-called vortex checkerboard, is nucleated in the vortex core^20^,^21^,^22^. Although a possible connection between the vortex checkerboard and the electronic order that breaks spatial symmetry has been discussed^20^,^21^,^22^, the electronic states in the vortex core are still elusive, probably because the energy ranges so far studied are mostly below Δ_0_ and little is known about the momentum-space electronic states in the vortex core.

Our Fourier-transform SI-STM study over a wide energy range has revealed bipartite electronic superstructures in the vortex core of Bi_2_Sr_2_CaCu_2_O_8+*δ*_. We show that the vortex checkerboard below Δ_0_ does not represent the electronic order but is associated with the *B*-enhanced BQPI pattern. New *B*-enhanced feature has been found at the pseudogap energy scale Δ_1_ where the electronic state is characterized by the broken-spatial-symmetry state^2^,^6^,^7^,^8^,^9^,^10^,^11^,^12^; the modulation amplitude of this electronic superstructure is locally enhanced in the vortex core. This consolidates the competitive relation between superconductivity and the broken-spatial-symmetry state associated with the pseudogap.

## Results

### Spectroscopic features around vortices

**Figure 1:**
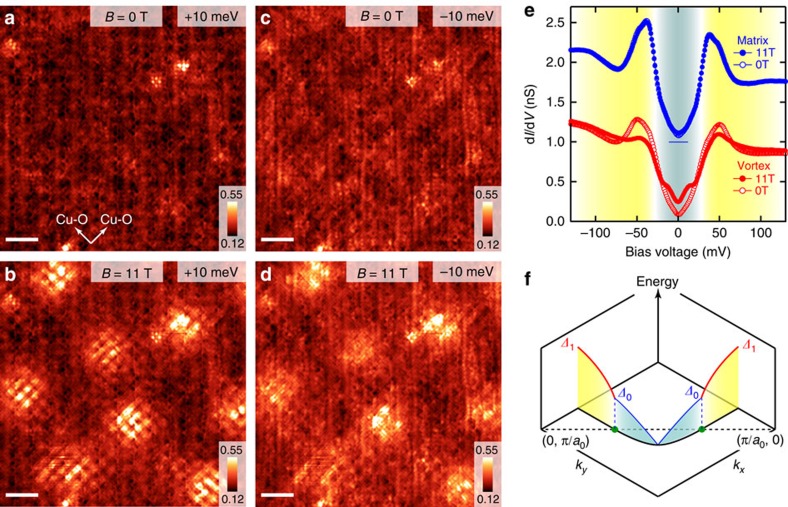
Spectroscopic features of vortices and nodal–antinodal dichotomy. (**a**,**b**) Differential conductance maps at energy *E*=+10 meV in magnetic fields *B*=0 and 11 T, respectively. White arrows in **a** denote the Cu–O bonding directions. Vortices and their internal structures (vortex checkerboard) are clearly imaged in **b**. (**c**,**d**) Differential conductance maps at *E*=−10 meV taken in *B*=0 and 11 T. Scale bars, 50 Å (**a**–**d**), and the colour scales are in nano siemens (nS). The tunnelling conductance at each location was obtained by numerical differentiation of the current–voltage characteristics and by post-smoothing with the energy window of ±2 meV. (**e**) Comparison between tunnelling spectra taken at *B*=0 T (open symbols) and 11 T (solid symbols). Red and blue data depict the spectra spatially averaged over the regions near vortices and far from vortices, respectively. (**f**) Schematic illustration of the excitation gap in momentum-space showing the dichotomy between the *d*-wave superconductivity near the node (light blue area) and the antinodal states governed by the pseudogap (yellow area). These two regimes are separated by the line connecting (*π*/*a*_0_, 0) and (0, *π*/*a*_0_), where *a*_0_ denotes Cu–O–Cu distance^1^,^2^.

Figure 1a–d show differential tunnelling conductance *g*(**r**, *E*, *B*) maps at energy *E*=±10 meV taken at *B*=0 and 11 T in exactly the same field of view. Here, **r** denotes the position on the surface. Vortex cores are identified as enhanced *g*(**r**, *E*, *B*) regions with the vortex checkerboard structure^20^,^21^,^22^. (Effects of *B* on various spectroscopic quantities are shown in Supplementary Fig. 1). Figure 1e represents the spectra spatially averaged over the regions near vortices (vortex region) and far from vortices (matrix region). (Definition of these regions are described in the ‘Methods' section and Supplementary Fig. 2). As shown in Fig. 1e, the vortex alters the spectrum in two different energy regions^21^,^22^,^23^,^24^,^25^: the emergence of conductance humps around 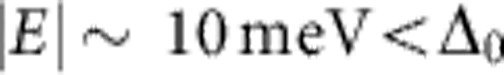 and the suppression of the peaks at Δ_1_. (Detailed point spectra in a single vortex core are shown in Supplementary Fig. 3). Since these two energy regions occupy different sectors in momentum-space, near-nodal and antinodal states (Fig. 1f), it is intriguing to explore the momentum-space characters of the vortex-induced electronic states.

By taking the Fourier transformation from the spectroscopic images, we can estimate the characteristic wavevectors **q**(*E*, *B*)'s of electronic-state modulations. However, in heterogeneous systems such as Bi_2_Sr_2_CaCu_2_O_8+*δ*_, *g* (**r**, *E*, *B*) not only reflects the **r** dependence of the local density-of-states (LDOS) at *E* but also includes LDOS modulations at different energies because of the **r**-dependent tip elevation associated with the feedback loop. This so-called set-point effect can be suppressed by taking a ratio *Z*(**r**,*E*, *B*)=*g* (**r**,+|*E*|,*B*)/*g* (**r**,−|*E*|,*B*), which faithfully represents the ratio of the LDOS at ±|*E*| (refs 6, 26).

### Effect of the vortex on low-energy electronic states

**Figure 2:**
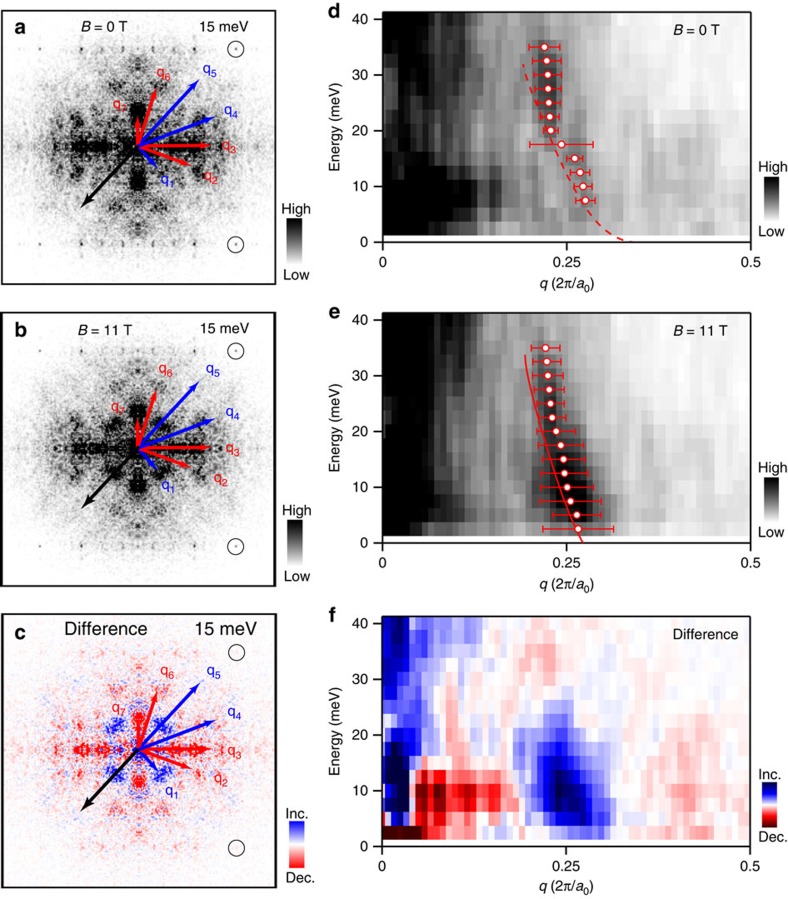
Bogoliubov quasiparticle interference origin of the vortex checkerboard. (**a**,**b**) Conductance-ratio maps in scattering-vector **q** space *Z*_*q*_(**q**, *E*, *B*) at energy *E*=15 meV in magnetic fields *B*=0 and 11 T, respectively. Each map was obtained by the Fourier transformation of the real-space conductance-ratio map taken with the field of view of 470 × 470 Å^2^, followed by the twofold symmetrization. The tunnelling conductance at each location was taken by the standard lock-in technique with a modulation amplitude of 2.5 mV_rms_. Red and blue arrows indicate the sign-reversing (**q**_2_, **q**_3_, **q**_6_, **q**_7_) and sign-preserving scattering (**q**_1_, **q**_4_, **q**_5_) wavevectors, respectively. Black circles show the Bragg spots. (**c**) Difference between a and b *Z*_*q*_(**q**, *E*=15 meV, *B*=11 T)−*Z*_*q*_(**q**, *E*=15 meV, *B*=0 T). (**d**,**e**) Energy-dependent line profiles taken along the black arrows in **a** and **b**, respectively. Red open circles denote the position of the **q**_1_ peak that has been determined by fitting the line profile at each energy by a Lorentzian function. Error bars indicate the full width at half maximum of the fitted peak. Dashed red line in **d** and solid red line in **e** are the dispersions calculated from **q**_2_, **q**_3_, **q**_6_ and **q**_7_ based on the octet model. Note that the *B*-induced signal exhibits the dispersion that is consistent with the Bogoliubov quasiparticle interference. (**f**) Energy-dependent line profile taken along the black arrow in **c**, showing the *B*-induced change.

First, we focus on the low-energy near-nodal region and argue the origin of the vortex checkerboard. At *B*=0 T, the only relevant phenomenon near the node is the BQPI that is described by the octet model^4^,^5^ in which the eight tips of the banana-shaped constant-energy contours in momentum-space dominate the quasiparticle scatterings, resulting in a set of energy-dispersive characteristic wavevectors **q**_*i*_ (*i*=1, 2,⋯, 7; Supplementary Fig. 4 and Supplementary Note 1). Figure 2a depicts the typical BQPI pattern seen in *Z*_*q*_(**q**, *E*, *B*=0 T), the Fourier-transformed image of *Z*(**r**, *E*, *B*=0 T), showing the octet **q**_*i*_'s. (In the optimally doped Bi_2_Sr_2_CaCu_2_O_8+*δ*_, signals at **q**_4_ and **q**_5_ are weak in *Z* (refs 2, 10). We have performed standard BQPI analysis (Supplementary Fig. 4 and Supplementary Note 1)^2^,^5^, and confirm that the BQPI is restricted in the near-nodal region below the extinction energy Δ_0_∼30 meV (Supplementary Fig. 5)^2^.

An important question here is whether the vortex checkerboard, which is most prominent around 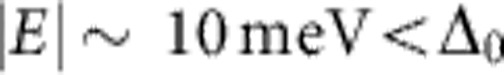, represents an electronic order or not. To answer this question, we have repeated the same *Z*(**r**, *E*, *B*) analysis at *B*=11 T in the same field of view (Supplementary Fig. 4 and Supplementary Note 1). As shown in Fig. 2b, no additional peak is detected, whereas the intensity of each **q**_*i*_ peak depends on *B*. Figure 2c highlights the *B*-induced change obtained by subtracting *Z*_*q*_(**q**, *E*, *B*=0 T) from *Z*_*q*_(**q**, *E*, *B*=11 T). The enhanced intensity appears at **q**_1_, which represents the wavevector of the vortex checkerboard. We note that the intensities at **q**_2_, **q**_3_, **q**_6_ and **q**_7_ are suppressed by *B* (Supplementary Fig. 5). All of these scattering **q**_*i*_'s reverse the sign of the *d*-wave superconducting gap between the initial state and the final state, while the sign is preserved in the the case of **q**_1_ scattering. Such suppression and enhancement of sign-reversing and sign-preserving scatterings, respectively, are exactly what are expected from the coherence factors of the quasiparticle scatterings off vortices^27^,^28^,^29^, suggesting that the cause of the vortex checkerboard is not the electronic order but the vortex-enhanced BQPI.

The BQPI scenario is further supported by the energy dispersion in the vortex-enhanced signal at **q**_1_ (Fig. 2d,e). The observed **q**_1_ dispersion agrees well with the behaviour calculated from **q**_2_, **q**_3_, **q**_6_ and **q**_7_ based on the octet model. Altogether with the fact that the vortex-enhanced signal diminishes near Δ_0_ (Fig. 2f and Supplementary Fig. 5), we ascribe the vortex checkerboard to the BQPI and conclude that no electronic order is nucleated in the vortex cores at *E*<Δ_0_. We note that other Friedel-type oscillations such as bound-state oscillations in the quantum-limit vortex core^22^ may also be relevant (Supplementary Note 2). In any case, our observation suggests that canonical phenomenology of *d*-wave superconductivity applies in the near-nodal region even when the vortices are introduced.

### Effect of the vortex on high-energy pseudogap states

**Figure 3:**
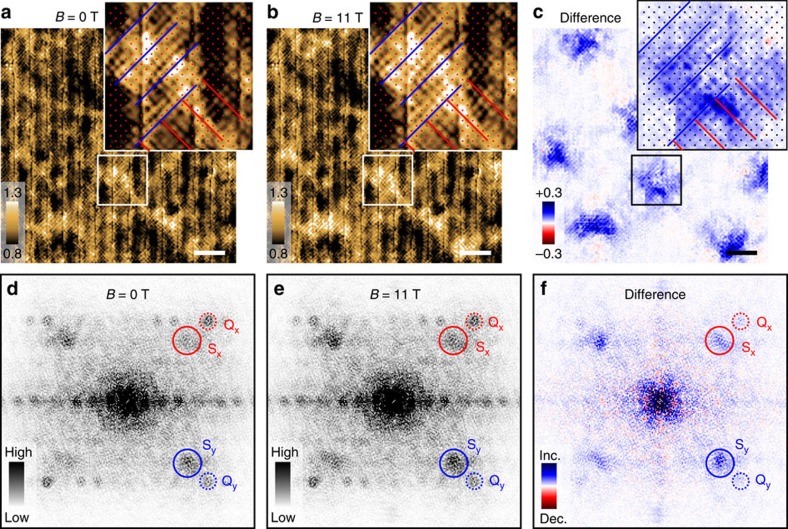
Magnetic field effect on the nanostripe at the pseudogap energy scale. (**a**,**b**) Conductance-ratio maps *Z*(**r**, *e*=1, *B*) at the position (**r**)-dependent pseudogap energy Δ_1_ in magnetic fields *B*=0 and 11 T, respectively. Here, *e*≡*E*/Δ_1_ where *E* denotes energy. The tunnelling conductance was obtained by numerical differentiation of the current–voltage characteristics and by post-smoothing with the energy window of ±10 meV. (**c**) Difference between (**a**,**b**) *Z*(**r**, *e*=1, *B*=11 T)−*Z*(**r**, *e*=1, *B*=0 T). Scale bars, 50 Å. Insets of **a**–**c** are the magnified images of the regions marked by boxes in the main figures. Red and blue lines in the insets indicate the directions of the nanostripe. Dots denote the locations of the Cu atoms. (**d**,**e**) Fourier-transformed images of **a** and **b**, respectively. The field of views are restricted in the vicinity of the vortices by applying a mask. (**f**) Difference between **d** and **e**. In these Fourier-transformed images, dotted and solid circles indicate the characteristic wavevectors of the nanostripe **Q**_*x*,*y*_=(2*π*/*a*_0_, 0),(0, 2*π*/*a*_0_) and **S**_*x,y*_∼3/4 × (2*π*/*a*_0_, 0), 3/4 × (0, 2*π*/*a*_0_), respectively.

It has been reported that the electronic feature above Δ_0_ is characterized by the bond-centered unidirectional electronic entity that breaks both rotational and translational symmetries^2^,^6^,^7^,^8^. For the purpose of brevity, we call this electronic entity as nanostripe hereafter. The nanostripe is reminiscent of the short-range charge order detected by X-ray scattering^30^,^31^ and may be responsible for the weakened energy dispersion of **q**_1_ near Δ_0_ (Fig. 2d,e)^32^. Here, we investigate the effect of *B* on the nanostripe. To visualize the nanostripe which is most prominent at Δ_1_, we follow the procedure used in (refs 2, 7). Since Δ_1_ is spatially inhomogeneous, we first normalize *E* by local Δ_1_(**r**) and map *Z*(**r**, *e*≡*E*/Δ_1_(**r**)=1, *B*). Figure 3a shows *Z*(**r**, *e*=1, *B*=0 T) in the same field of view as Fig. 1a–d. The nanostripe in the optimally doped sample is weak in intensity^10^ and is observed only in the limited regions where *Z*(**r**, *e*=1, *B*) is larger. We find that these regions have larger Δ_1_(**r**) and vortices tend to reside there, suggesting a vortex pinning mechanism associated with the pseudogap (Supplementary Fig. 6 and Supplementary Note 3). We have repeated the same measurement at *B*=11 T (Fig. 3b) and have revealed that *Z*(**r**, *e*=1, *B*) is enhanced in the vortex cores (Fig. 3c). As shown in the insets of Fig. 3a–c, the structure of the nanostripe that pre-exists in the absence of the vortex core is unchanged but its contrast is enhanced. The local enhancement of nanostripe in the vortex core has also been observed in (ref. 33). We note that the local nature of the enhancement indicates that the enhancement is not caused by *B* itself, which is almost uniform at 11 T because of the long penetration depth. Instead, it should be associated with the local suppression of superconductivity in the vortex core, representing the direct competition between superconductivity and the nanostripe.

It has been known that the nanostripe consists of two sets of wavevectors^7^: **Q**_*x*,*y*_=(2*π*/*a*_0_,0), (0,2*π*/*a*_0_) whose inequivalent intensities represent the degree of broken rotational symmetry and **S**_*x,y*_∼(3/4 × (2*π*/*a*_0_), 0), (0, 3/4 × (2*π*/*a*_0_)) that feature the broken translational symmetry. To test which broken symmetry is affected in the vortex core, we perform the Fourier analysis. By applying a mask generated from the image of vortices, we restrict our field of view in the vicinity of the vortex core to effectively extract the vortex-enhanced features (see the ‘Methods' section and Supplementary Fig. 2). As shown in Fig. 3d,e, Fourier peaks corresponding to **Q**_*x*,*y*_ and **S**_*x,y*_ are identified at both *B*=0 and 11 T. In the difference image shown in Fig. 3f, the intensity at **S**_*x,y*_ is enhanced, whereas the change at **Q**_*x*,*y*_ is small. This observation indicates that the vortex core predominantly amplifies the translational symmetry breaking that is associated with the pseudogap (Supplementary Fig. 7 and Supplementary Note 4). The vortex-enhanced nanostripe at *E*∼Δ_1_ is reminiscent of the *B*-enhanced charge orders in Y-based cuprates^15^,^16^,^17^,^18^,^19^, but further studies are necessary to make clear the similarities and differences between the *B*-induced charge orders in different materials.

## Discussion

It is noteworthy to argue the spectroscopic features in the vortex core where the spectral weights at 
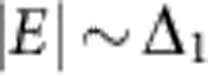
 and 
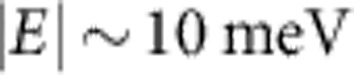
 are suppressed and enhanced, respectively (Fig. 1e). Given the close correlation between the pseudogap and the nanostripe (Supplementary Fig. 7 and Supplementary Note 4)^2^,^6^,^7^,^8^,^9^,^10^,^11^, the missing weight at the pseudogap energy Δ_1_ along with the enhanced nanostripe demands careful consideration. It may be possible that the enhanced nanostripe causes the extra quasiparticle decoherence^34^ that suppresses the weight at 
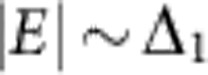
. However, this scenario can not account for the new states created at 
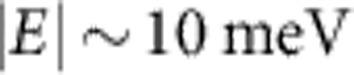
. Here, we point out that the observed spectral weight transfer from 
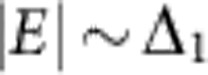
 to 
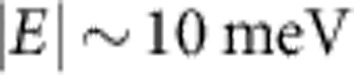
 can naturally be explained if the superconducting gap is as large as Δ_1_; the missing weight at 
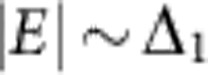
 is originated from the suppression of superconductivity, being transfered to the vortex-bound states at 
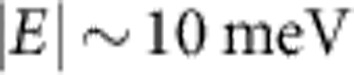
 (refs 22, 35). Since two different orders, in the present case, superconductivity and the nanostripe, can not share the same region in energy-momentum-space, it is plausible that they are nearly degenerated but competing states near the antinode^15^,^19^,^35^,^36^.

This scenario provides an insight into the heterogeneous electronic states in real-space. Randomly-dispersed dopants and defects locally perturb the balance between superconductivity and the nanostripe, and consequently bring about the nano-scale mixture of them. The observed vortex-enhanced nanostripe suggests that the vortex core is one of these perturbations that affects the competition near the antinode. This competition scenario of the *B*-enhanced electronic order is based on our unambiguous identification of the energy scale of the phenomenon and can be further tested by controlling the energy scales of superconductivity and the pseudogap by doping.

## Methods

### SI-STM measurements and sample preparation

SI-STM experiments were performed at a temperature of 4.6 K with a modified commercial low-temperature ultra-high-vacuum STM (Unisoku USM-1300) installed in RIKEN^37^. Single crystals of Bi_2_Sr_2_CaCu_2_O_8+*δ*_ were grown at the University of Tokyo by the traveling-solvent floating-zone method, and were annealed to have optimal hole concentration. The superconducting transition temperature *T*_*c*_∼90 K was determined by the magnetization measurement. The sample was cleaved *in situ* at ∼77 K to obtain a clean and flat (001) surface and was transferred quickly to the STM unit kept at 4.6 K. We used an electro-chemically etched tungsten wire as an STM tip, which was cleaned and characterized *in situ* with a field-ion microscope. All the SI-STM data were taken with the feedback set point at a sample bias voltage of −150 mV and a tunnelling current of 150 pA. *B* was applied perpendicular to the cleaved (001) surface. Whenever we changed *B*, the sample was heated up to ∼30 K to make the vortex distribution inside the sample uniform.

### Procedure to correct image distortions

It is well-known that electronic states of Bi_2_Sr_2_CaCu_2_O_8+*δ*_ are heterogeneous especially near Δ_1_. Therefore, to argue the vortex-induced local changes in the electronic state, it is indispensable to compare two SI-STM data sets, with and without a magnetic field, in exactly the same field of view. This is challenging because the actual SI-STM images are inevitably distorted in an uncontrollable manner due to the creeping of the piezoelectric scanner and so on. To obtain distortion-free images from the observed ones, we utilize the so-called Lawler–Fujita algorithm^7^, which estimates the local distortion as a phase shift in the crystal-lattice modulations. We first estimate and correct the distortions in the topographic images simultaneously taken with the *g* (**r**, *E*, *B*) images and correct the spectroscopic images, *g* (**r**, *E*, *B*) and *Z* (**r**, *E*, *B*), using the same local distortions. Various images obtained by SI-STM after the correction are shown in Supplementary Fig. 1.

### Definition of the vortex and matrix regions

Vortices are most clearly seen in the difference map *δg* (**r**, *E*=+10 meV, *B*=11 T)≡*g* (**r**, *E* =+10 meV, *B*=11 T)−*g* (**r**, *E*=+10 meV, *B*=0 T) (Supplementary Fig. 2a). We first apply low-pass filter to the *δg* (**r**, *E*=+10 meV, *B*=11 T) map with a cutoff wavelength of *q*∼0.05 × 2*π*/*a*_0_. Constant contours of this filtered image are used for the boundaries of the masks (Supplementary Fig. 2b). The vortex and matrix regions used to examine the vortex-induced change are shown in Supplementary Fig. 2c. The spectra shown in Fig. 1e are the spectra averaged in these regions. In Supplementary Fig. 3, we depict the detailed point spectra near the vortex before averaging. The mask used for the Fourier analyses (Fig. 3d–f) and the restricted *Z* (**r**, *E*, *B*) maps at *B*=0 and 11 T are indicated in Supplementary Fig. 2d–f, respectively.

### Data availability

All relevant data are available on request, which should be addressed to T.M. or T.H.

## Additional information

**How to cite this article:** Machida, T. *et al*. Bipartite electronic superstructures in the vortex core of Bi_2_Sr_2_CaCu_2_O_8+*δ*_. *Nat. Commun.* 7:11747 doi: 10.1038/ncomms11747 (2016).

## Supplementary Material

Supplementary Information Supplementary Figures 1-7, Supplementary Notes 1-4 and Supplementary References

Peer Review File
