# Supplementary material for: Bipartite electronic superstructures in the vortex core of Bi2Sr2CaCu2O8+δ
Source: Nat Commun. 2016 May 27;7:11747. doi: 10.1038/ncomms11747 (PMC4895017; doi:10.1038/ncomms11747)
Supplement: Supplementary Information — Supplementary Figures 1-7, Supplementary Notes 1-4 and Supplementary References [file ncomms11747-s1.pdf]

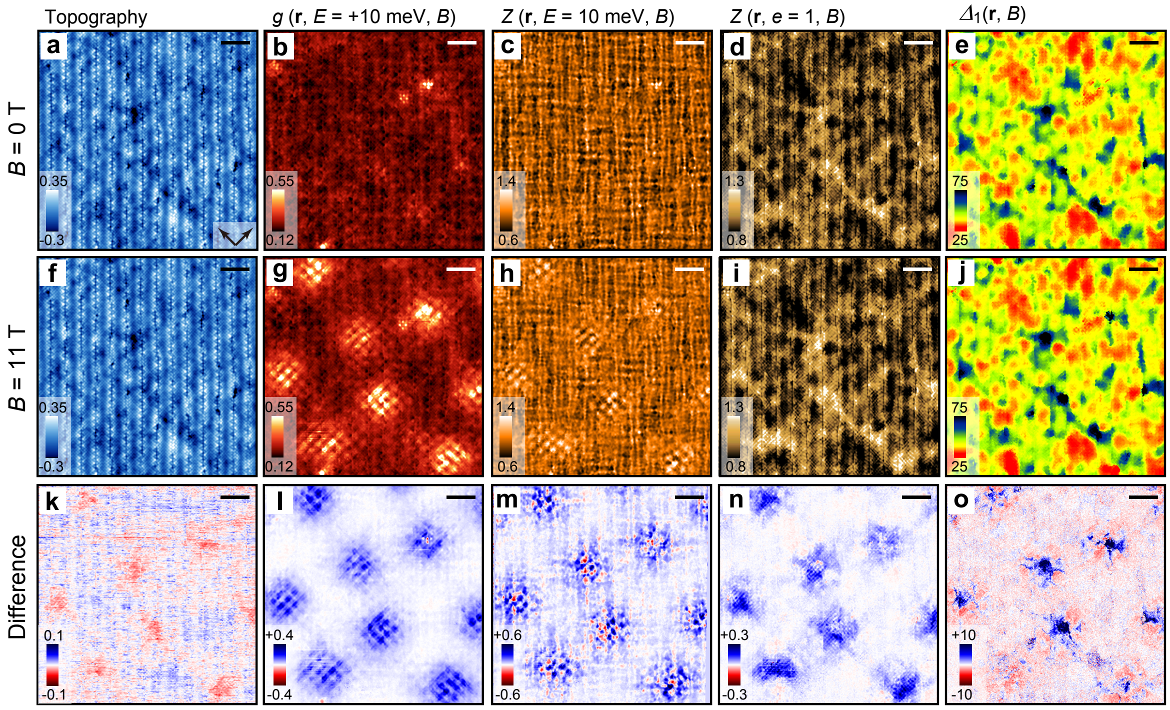

Supplementary Figure 1: **Effects of magnetic field on various quantities.** Various real-space **r** images taken in the same 380 × 380 Å^2 area. (**a-e**) Topographic image, differential conductance map $g(\mathbf{r}, E, B)$ at energy $E = +10$ meV, conductance ratio map $Z(\mathbf{r}, E = 10 \text{ meV}, B)$, conductance ratio map $Z(\mathbf{r}, e = 1, B)$ at the pseudogap energy Δ_1 ($e \equiv E/\Delta_1$) and the gap map $\Delta_1(\mathbf{r}, B)$, respectively, in the absence of magnetic field B. The scale bars correspond to 50 Å. The color scales are in Å for **a**, in nS for **b**, in arbitrary units for **c** and **d**, and in meV for **e**. Black arrows in **a** denote the Cu-O bonding directions. (**f-j**) Same as (**a**) to (**e**) except that $B = 11$ T. (**k-o**) Difference images obtained by subtracting the results at $B = 0$ T from those at $B = 11$ T. Although an apparent B-induced enhancement of $\Delta_1(\mathbf{r}, B)$ is observed in some of the vortex cores, changes in the original $g(\mathbf{r}, E, B)$ spectra near $\Delta_1(\mathbf{r}, B)$ are very small; due to small $dg(\mathbf{r}, E, B)/dE$ at $E \sim \Delta_1(\mathbf{r}, B)$, small change in $g(\mathbf{r}, E, B)$ gives rise to large effect on the estimation of $\Delta_1(\mathbf{r}, B)$. We have used $\Delta_1(\mathbf{r}, B = 0 \text{ T})$ to create both of $Z(\mathbf{r}, e = 1, B = 0 \text{ T})$ and $Z(\mathbf{r}, e = 1, B = 11 \text{ T})$ maps.

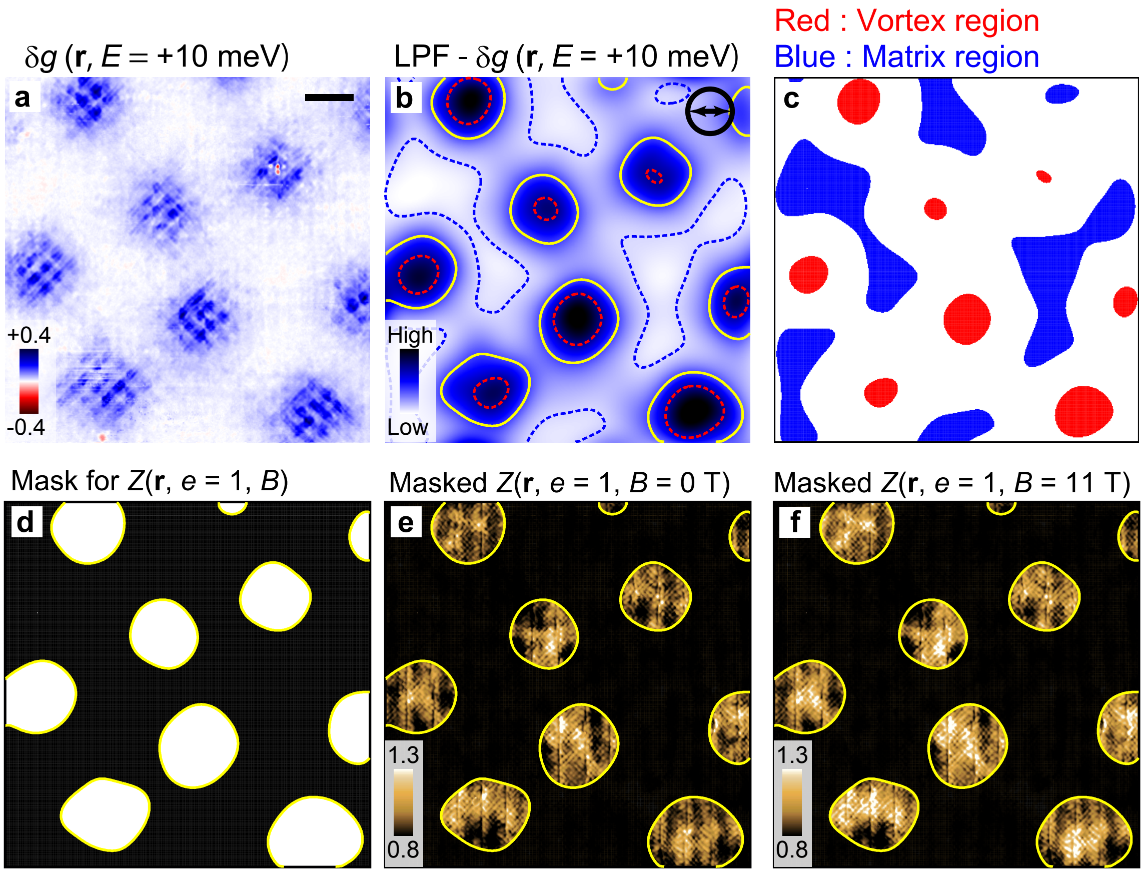

Supplementary Figure 2: **Definition of vortex and matrix regions.** (**a**) Difference between the two conductance g maps in real space $\mathbf{r}$ at magnetic field $B = 0$ T and 11 T; $\delta g(\mathbf{r}, E = +10 \text{ meV}) \equiv g(\mathbf{r}, E = +10 \text{ meV}, B = 11 \text{ T}) - g(\mathbf{r}, E = +10 \text{ meV}, B = 0 \text{ T})$, where E denotes energy. The scale bar corresponds to 50 Å. The color scale is in nS. (**b**) Low-pass filtered image of **a**; LPF-$\delta g(\mathbf{r}, E = +10 \text{ meV})$. A cut-off wavevector is chosen to be $0.05 \times 2\pi/a_0$ in the filtering process, where a_0 denotes Cu-O-Cu distance. Corresponding spatial resolution is indicated by a black circle and an arrow. Red dashed, blue dashed, and yellow lines are the contours with the value of 65, 8, 35% of the difference between the maximum and minimum values of this image, respectively. (**c**) Vortex and matrix regions in which the spectra are spatially averaged for comparison. Red and blue regions denote vortex and matrix regions and are defined as the regions surrounded by the red and blue dashed lines in **b**. (**d**) A mask used to extract the field-effect on the nanostripe that appears in the conductance ratio map $Z(\mathbf{r}, e = 1, B)$ at the pseudogap energy Δ_1 ($e \equiv E/\Delta_1$). White and black regions denote the vortex and matrix regions, which are separated by the yellow contours in **b**. (**e**, **f**) Masked conductance ratio maps at $e = 1$ in $B = 0$ T and 11 T, respectively.

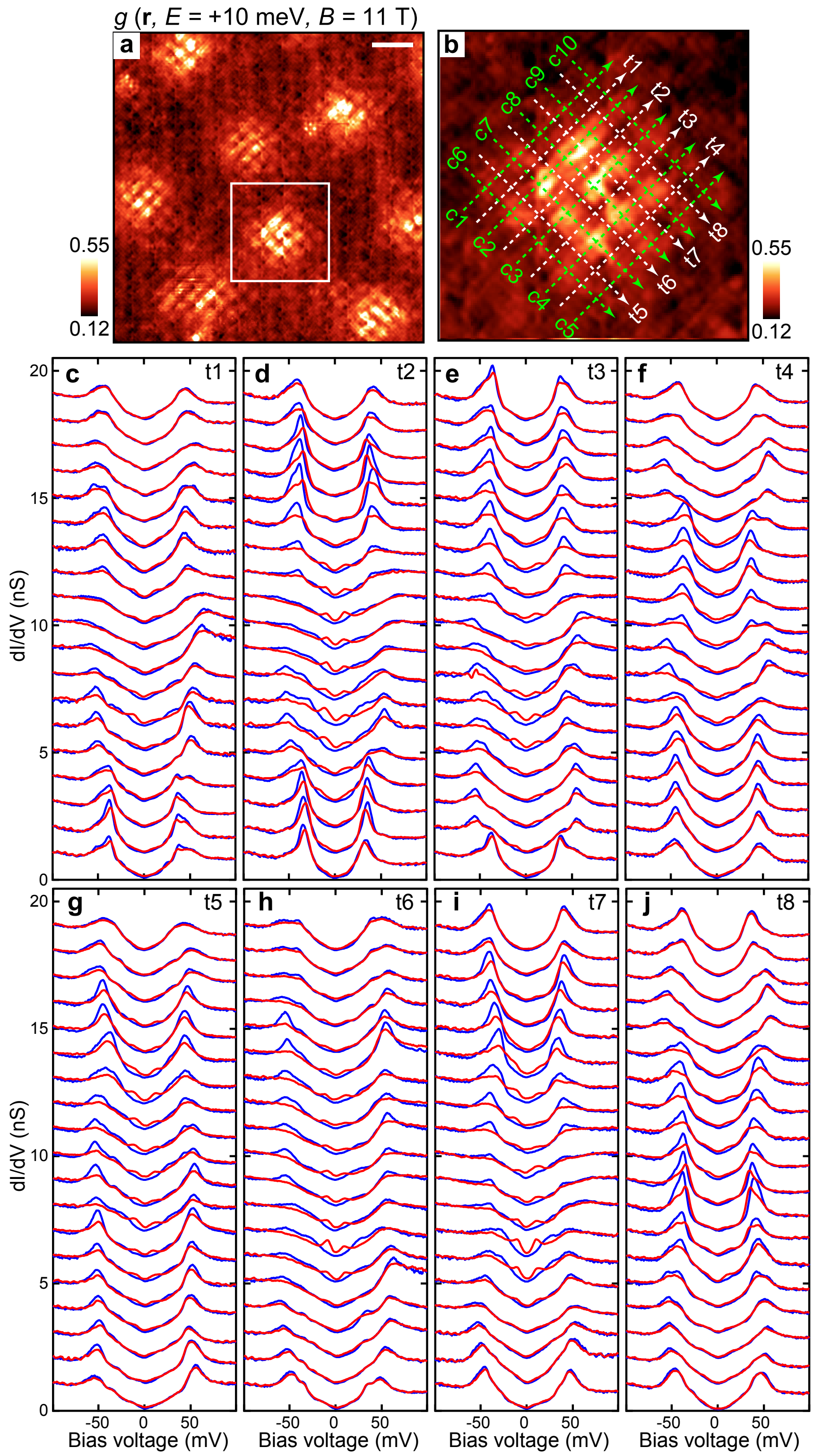

Supplementary Figure 3: **Spatial variation of tunneling spectra around a single vortex core.** Continued on next page.

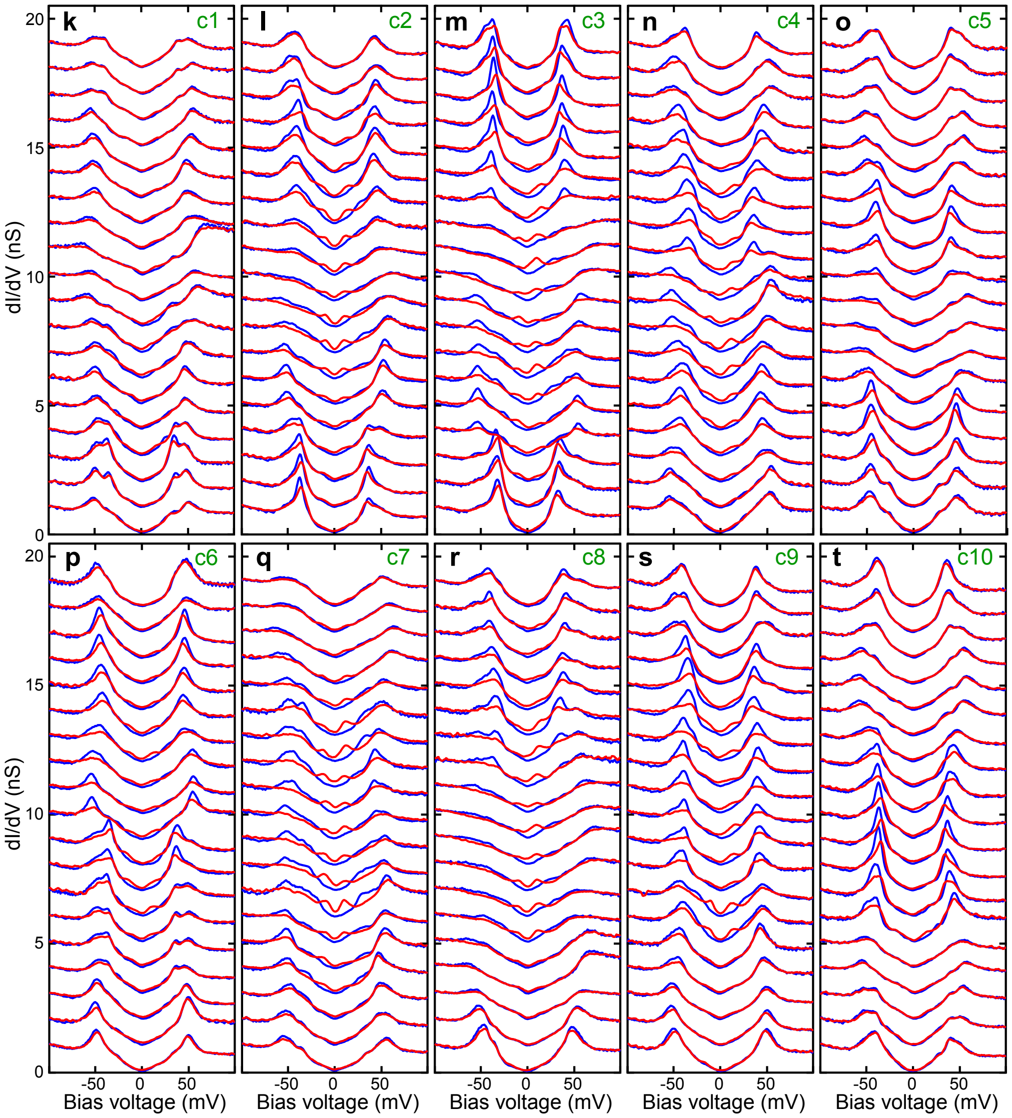

Supplementary Figure 3: **Spatial variation of tunneling spectra around a single vortex core.** (**a**) Differential conductance map g in real space $\mathbf{r}$ at energy $E = +10$ meV and magnetic field $B = 11$ T. The scale bar corresponds to 50 Å. (**b**) Magnified image of the area marked by a white box in **a**. The color scales of **a** and **b** are in nS. Tunneling spectra taken along 8 troughs (**c**-**j**) and 10 crests (**k**-**t**) of the vortex checkerboard shown in **b**. Each spectrum is offset for clarity. Bottom most spectra correspond to the starting points of the arrows drawn in **b**. Blue and red curves denote the tunneling spectra at $B = 0$ T and 11 T, respectively.

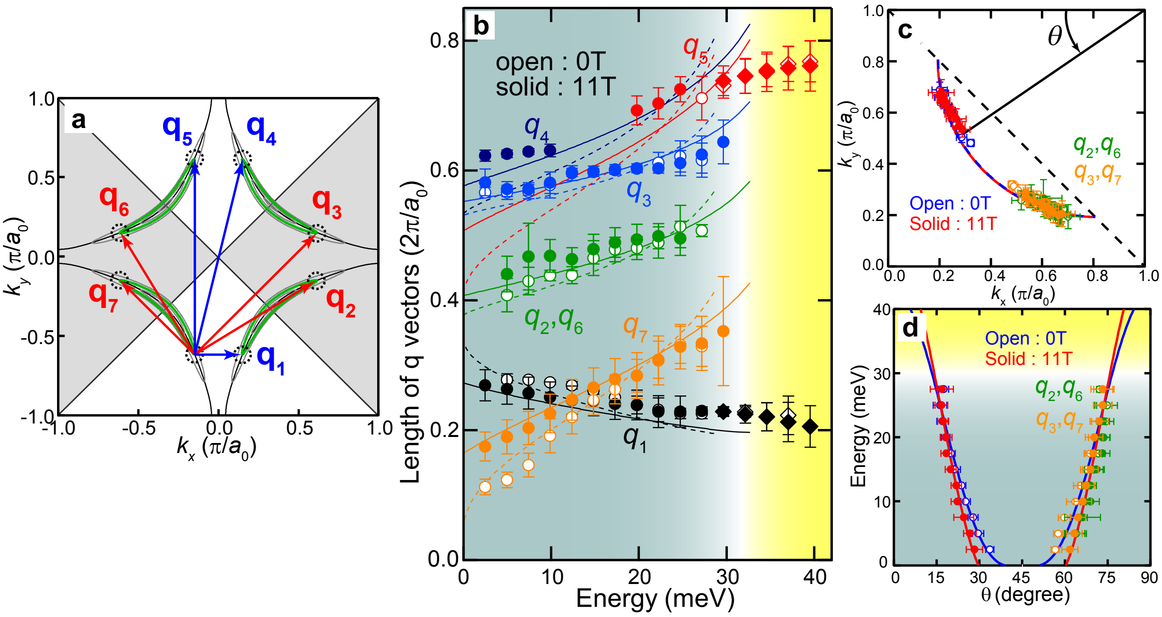

Supplementary Figure 4: **Bogoliubov quasiparticle interference analyses based on the octet model.** (**a**) Schematic illustration of the momentum-space electronic structure. White and gray areas indicate the regions with the opposite signs of the d-wave superconducting gap. Black and gray solid lines represent normal-state Fermi surface and constant energy contours of the Bogoliubov quasiparticle dispersion. Green bold lines denote constant energy contours at a representative energy. Black dashed circles correspond to the eight high density-of-states regions, which primarily contribute the Bogoliubov quasiparticle interference (BQPI) at a given energy E. Red and blue arrows indicate sign-reversing and sign-preserving scattering vectors, respectively [1]. (**b**) Energy dependence of the absolute value of the observed wavevectors $\mathbf{q}_i$'s at magnetic field $B = 0$ T (open symbols) and 11 T (solid symbols). Error bars denote the average of two full widths at half maxima (σ_x and σ_y) of the fitted two-dimensional Lorentzian functions. Dashed and solid lines denote the expected dispersions at $B = 0$ T and 11 T, respectively. These dispersions are determined from pairs of ($\mathbf{q}_2$, $\mathbf{q}_6$) and ($\mathbf{q}_3$, $\mathbf{q}_7$). (**c**) Fermi surface loci obtained from BQPI peak locations $\mathbf{q}_i(E)$, at $B = 0$ T (open symbols) and 11 T (solid symbols). Locations of green and orange circles are obtained from pairs of ($\mathbf{q}_2$, $\mathbf{q}_6$) and ($\mathbf{q}_3$, $\mathbf{q}_7$), respectively. Blue and red circles represent the average of them. Error bars are estimated from the full widths at half maxima of the fitted two-dimensional Lorentzian functions using the error propagation formula. Blue dashed and red solid lines denote the results of quarter circle fitting. (**d**) Fermi-surface angle θ dependence of the superconducting gap at $B = 0$ T (blue symbols) and 11 T (red symbols). Data shown by green and orange circles are estimated from pairs of ($\mathbf{q}_2$, $\mathbf{q}_6$) and ($\mathbf{q}_3$, $\mathbf{q}_7$), respectively. Blue and red circles represent the average of them. These data are fitted to $\Delta(\theta) = \Delta_{\rm BQPI}[A\cos(2\theta) + (1-A)\cos(6\theta)]$ as shown by the solid curves. Here, $\Delta(\theta)$ and $\Delta_{\rm BQPI}$ denote the gap amplitude at θ and the hypothetical maximum energy of the Bogoliubov quasiparticle excitation, respectively.

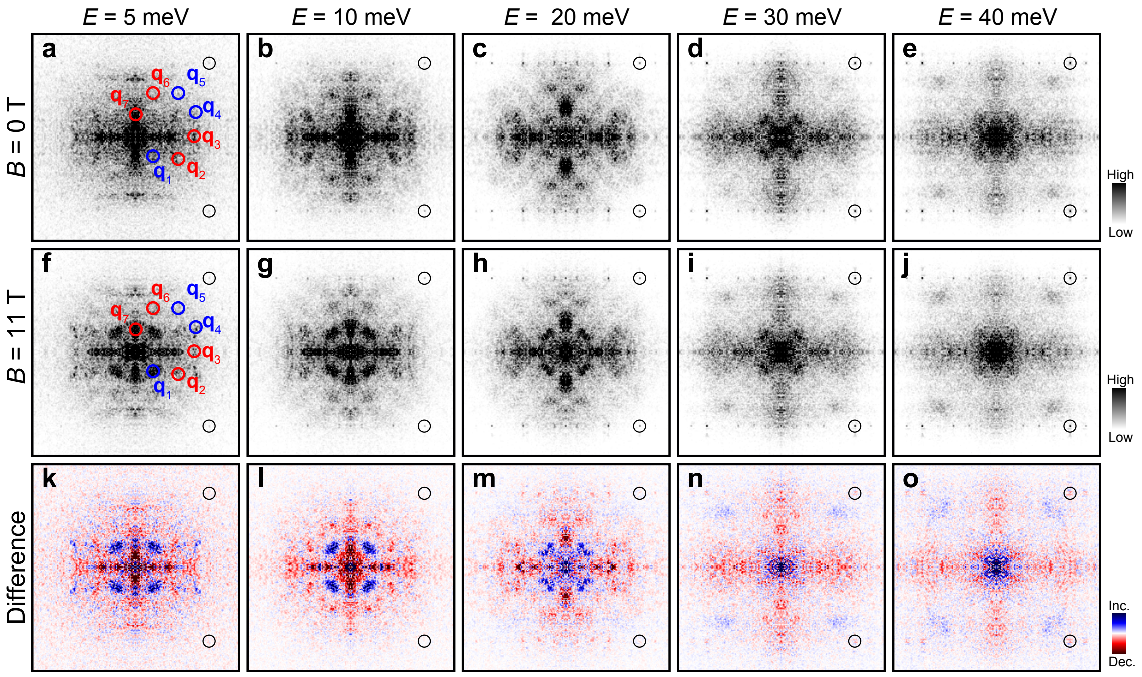

Supplementary Figure 5: **Extinction of the Bogoliubov quasiparticle interference pattern at high energies.** (**a-e**) Conductance-ratio maps in scattering-vector **q** space $Z_q(\mathbf{q}, E, B)$ in the absence of magnetic field B at energies $E = 5$, 10, 20, 30, and 40 meV, respectively. (**f-j**) Same as **a-e**, except that $B = 11$ T. (**k-o**) Difference images, which are obtained by subtracting the results at $B = 0$ T from those at $B = 11$ T. Clear Bogoliubov quasiparticle interference patterns diminish above $E \sim 30$ meV.

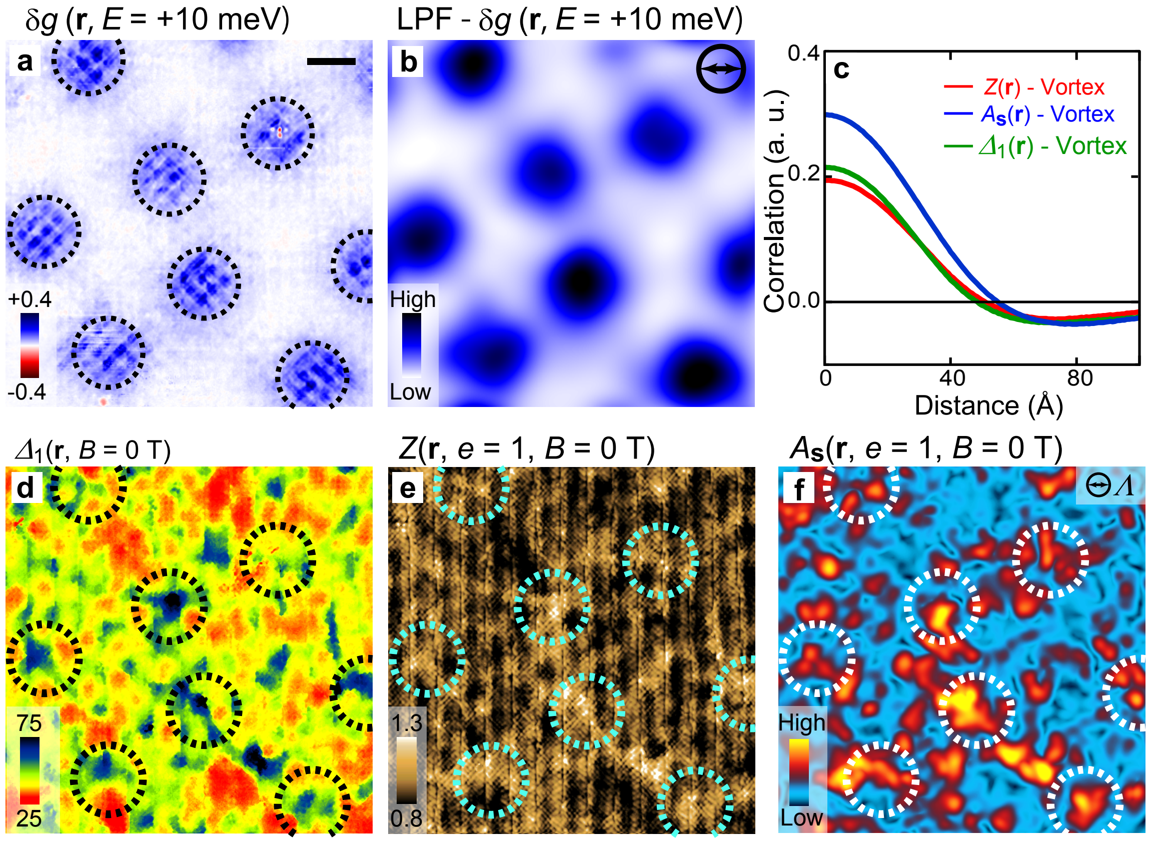

Supplementary Figure 6: **Spatial correlations between vortices and quantities associated with the pseudogap.** (**a**) Difference between the two conductance maps g in real space $\mathbf{r}$ taken at energy $E = +10$ meV in magnetic fields $B = 0$ T and 11 T; $\delta g(\mathbf{r}, E, B = 11\ \text{T}) \equiv g(\mathbf{r}, E, B = 11\ \text{T}) - g(\mathbf{r}, E, B = 0\ \text{T})$. Black dashed circles mark the vortices. The scale bar corresponds to 50 Å. The color scale is in nS. (**b**) Low-pass filtered image of **a**; LPF-$\delta g(\mathbf{r}, E = +10\ \text{meV})$. A cut-off wavevector is chosen to be $0.05 \times 2\pi/a_0$ in the filtering process, where a_0 denotes Cu-O-Cu distance. Corresponding spatial resolution is indicated by a black circle and an arrow. (**c**) Azimuthally averaged cross-correlations for pairs of [LPF-$\delta g(\mathbf{r})$, $\Delta_1(\mathbf{r})$] (Green curve), and [LPF-$\delta g(\mathbf{r})$, $Z(\mathbf{r})$] (Red curve), and [LPF-$\delta g(\mathbf{r})$, $A_\text{S}(\mathbf{r})$] (Blue curve). Here, $\Delta_1(\mathbf{r})$ is the gap map in $B = 0$ T (**d**), $Z(\mathbf{r})$ is the conductance ratio map at $E = \Delta_1(\mathbf{r})$ ($e \equiv E/\Delta_1$=1) in $B = 0$ T (**e**) and $A_\text{S}(\mathbf{r})$ denotes the spatial variation of the nanostripe amplitude at $e = 1$ in $B = 0$ T (**f**). We define the nanostripe amplitude as the sum of the local amplitudes of modulations at the wavevectors $\mathbf{S}_x \sim 3/4 \times (2\pi/a_0, 0)$ and $\mathbf{S}_y \sim 3/4 \times (0, 2\pi/a_0)$. Dashed circles in these figures represent the locations of the vortices. Note that vortices tend to be pinned at the locations with large $\Delta_1(\mathbf{r})$, $Z(\mathbf{r})$, and $A_\text{S}(\mathbf{r})$ in the absence of B.

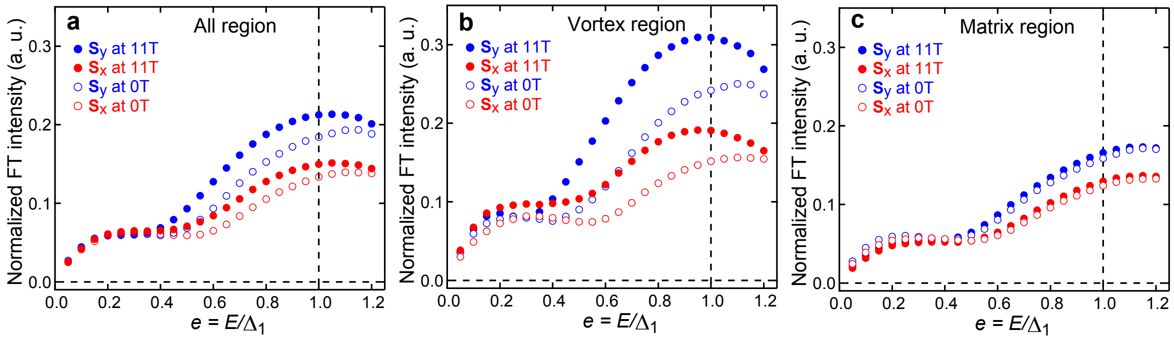

Supplementary Figure 7: **Nanostripe intensity maximizes at the pseudogap energy.** Normalized energy e dependence of the nanostripe intensities in magnetic fields $B = 0$ T and 11 T. Here, $e \equiv E/\Delta_1$, where E and Δ_1 are energy and the pseudogap energy, respectively. The nanostripe intensities are defined as the Fourier amplitudes at the wavevectors $\mathbf{S}_x \sim 3/4 \times (2\pi/a_0, 0)$ and $\mathbf{S}_y \sim 3/4 \times (0, 2\pi/a_0)$ in the conductance ratio map in scattering-vector $\mathbf{q}$ space $Z_q(\mathbf{q}, e, B)$. Here, a_0 denotes Cu-O-Cu distance. We performed analyses in different regions; all region (**a**), near vortices (**b**) and matrix region (**c**). Intensities are normalized according to the areas of the corresponding regions. Nanostripe intensity takes maximum at $e = 1$ and is enhanced by B in the vortex region.

Supplementary Note 1 - BQPI analyses based on the octet model

BQPI brings about LDOS modulations which are reflected on $g(\mathbf{r}, E, B)$. However, in actual fact $g(\mathbf{r}, E, B)$ may also contain extrinsic modulations caused by the set-point effect [2,3]. The set-point effect can be largely suppressed in $Z(\mathbf{r}, E, B)$, which we utilize throughout this work. The analysis of $Z(\mathbf{r}, E, B)$ has another advantage in that $Z(\mathbf{r}, E, B)$ picks up the particular modulations in which $\mathbf{q}(+|E|) = \mathbf{q}(-|E|)$ and the phase difference between the $\mathbf{q}(+|E|)$ and $\mathbf{q}(-|E|)$ modulations is large ($\sim \pi$). These features are exactly what are expected in the BQPI [3,4].

We use the octet model to analyze the BQPI seen in $Z_q(\mathbf{q}, E, B)$; the Fourier transformed $Z(\mathbf{r}, E, B)$ [5–7]. Here, the seven scattering vectors $\mathbf{q}_i$ (i = 1, 2,$\cdots$, 7) connecting the eight tips of the banana-shaped constant contours in momentum space govern the BQPI because the joint density of states takes maximum for these wavevectors (Supplementary Fig. 4a). As shown in Fig. 2a,b of the main text and in Supplementary Fig. 5, a set of energy-dispersive wavevectors are detected in $Z_q(\mathbf{q}, E, B)$ and each of the wavevectors can be assigned to one of the octet wavevectors $\mathbf{q}_i$. The intensities at $\mathbf{q}_4$ and $\mathbf{q}_5$ are weak, being consistent with the previous reports [8,9].

To determine the precise locations of $\mathbf{q}_i$'s at $B = 0$ T and 11 T, we fit the peaks in $Z_q(\mathbf{q}, E, B)$ with the two-dimensional Lorentzian function plus linear background;

$$f(q_x, q_y) = f_0 + \frac{A}{\left(\frac{q_x - q_{x0}}{\sigma_x}\right)^2 + \left(\frac{q_y - q_{y0}}{\sigma_y}\right)^2 + 1} + c_x q_x + c_y q_y, \qquad (1)$$

where f_0, c_x, and c_y are the fitting parameters associated with the linear background, whereas A, q_{x0}, q_{y0}, σ_x, and σ_y are the fitting parameters corresponding to the amplitude, the q_x and q_y components of the peak location, and the q_x and q_y components of the half width at half maximum of the peak, respectively. Supplementary Figure 4b shows the energy dependence of the absolute value of the observed $\mathbf{q}_i$'s. The signals at $\mathbf{q}_2$, $\mathbf{q}_3$, $\mathbf{q}_6$, and $\mathbf{q}_7$ diminishes at about 30 meV, which set the extinction energy Δ_0 (Supplementary Fig. 4b,d, Supplementary Fig. 5). At $E > \Delta_0$, signals at $\mathbf{q}_1$ and $\mathbf{q}_5$ are still there but lose their energy dependence. The signal near $\mathbf{q}_5$ turns into $\mathbf{S}$, which is one of the ingredients of the nanostripe.

We obtain the normal-state Fermi surface and the superconducting gap dispersion using pairs of ($\mathbf{q}_2$, $\mathbf{q}_6$) and ($\mathbf{q}_3$, $\mathbf{q}_7$). Supplementary Figure 4c depicts the Fermi-surface loci that sustain coherent Bogoliubov quasiparticles. They are limited inside the diagonal line connecting (π/a_0,0) and (0, π/a_0) [8] and are hardly affected by a magnetic field. By contrast a magnetic field suppresses the near-nodal superconducting gap as shown in Supplementary Fig. 4d. These features are consistent with the behaviors observed in a different cuprate superconductor $Ca_{2-x}Na_xCuO_2Cl_2$ [1] and can be associated with the Volovik effect [10].

In order to check the validity of the octet model especially in a magnetic field, we have performed the following analyses. First, we fit the Fermi-surface loci to the quarter circle. Next, the superconducting gap dispersions $\Delta(\theta)$ are fitted by the following d-wave form with an higher-order term.

$$\Delta(\theta) = \Delta_{\mathrm{BQPI}}[A\cos(2\theta) + (1 - A)\cos(6\theta)]. \qquad (2)$$

Here, θ represents the Fermi-surface angle defined in Supplementary Fig. 4c and Δ_{BQPI} and A are fitting parameters. Using the obtained fitting parameters, the energy dispersions of all the $\mathbf{q}_i$'s can be calculated and plotted in Supplementary Fig. 4b and in Fig. 2d,e of the main text. The vortex checkerboard is characterized by the field-enhanced signal at $\mathbf{q}_1$. We note that the energy dispersion of $\mathbf{q}_1$ at $B = 11$ T well coincides with the calculated one, as in the case at $B = 0$ T. Together with the fact that the $\mathbf{q}_4$ and $\mathbf{q}_5$ are also consistent with the calculated dispersions, we conclude that the field-induced change in the electronic state can be explained in the framework of the octet model as long as $E < \Delta_0$.

Supplementary Note 2 - Possible origins of the enhanced $\mathbf{q}_1$ modulations

The applicability of the octet model implies that the observed electronic-state modulations are associated with the Bogoliubov quasiparticles that reside on the near-nodal Fermi surface. There is more than one origin to cause such Friedel-type oscillations. Most naively, the enhanced quasiparticle scattering off vortices may result in the enhanced $\mathbf{q}_1$ [1]. Another possible origin is the spatial oscillations of the vortex bound state in the quantum-limit vortex core [11], as proposed by Yoshizawa and coworkers [12]. This model

naturally explains the peaks in the spectrum at $\sim \pm 10$ meV as the discrete bound states. Nevertheless, more studies are necessary to verify the validity of the bound-state scenario, because it is not clear whether the vortex of a cuprate is in the quantum limit and the formation of the vortex bound state in a d-wave superconductor is still a controversial issue [13,14].

Supplementary Note 3 - Vortex pinning and the competition between the nanostripe and superconductivity

It is interesting to examine the relationship between the locations of vortices and the various electronic heterogeneities, since this comparison may give us a hint to identify the elementary process of the vortex pinning. Although previous SI-STM studies have suggested that vortices in $Bi_2Sr_2CaCu_2O_{8+\delta}$ tend to be pinned at the regions where the $\Delta_1(\mathbf{r})$ is large [12,15], these experiments were conducted only in a field and thereby could not exclude the possibility that the the pseudogap itself would be influenced by vortices. Here we compare the locations of vortices with the zero-field electronic heterogeneity at $\Delta_1(\mathbf{r})$.

Using the same procedure described in Methods section in the main text, we first identify the locations of vortices (Supplementary Fig. 6a,b). We examine the azimuthally averaged cross-correlation function (Supplementary Fig. 6c) between the smoothed vortex map shown in Supplementary Fig. 6b and three different spectroscopic images at $\Delta_1(\mathbf{r})$: spatial variation of $\Delta_1(\mathbf{r})$ itself (Supplementary Fig. 6d), $Z(\mathbf{r}, e = 1, B)$ (Supplementary Fig. 6e), and the local amplitude of the broken-translational-symmetry state of the nanostripe, which is nothing but the local amplitude of the modulations at $\mathbf{S}_{x,y}$ (Supplementary Fig. 6f). Here, we define the local amplitude of these modulations $A_{\mathrm{S}}(\mathbf{r})$ as follows,

$$\begin{aligned} A_{\mathrm{S}}(\mathbf{r}) &\equiv A(\mathbf{S}_x, \mathbf{r}) + A(\mathbf{S}_y, \mathbf{r}), \\ A(\mathbf{S}_\nu, \mathbf{r}) &\equiv \sum_{\mathbf{r}'} Z(\mathbf{r}') e^{i\mathbf{S}_\nu \cdot \mathbf{r}} f_\Lambda(\mathbf{r}' - \mathbf{r}) \\ &\approx \frac{1}{\sqrt{N}} \sum_{\mathbf{k}} \tilde{Z}(\mathbf{S}_\nu - \mathbf{k}) e^{i\mathbf{k}\cdot\mathbf{r}} e^{-\mathbf{k}^2/2\Lambda^2} \qquad (\nu = x \text{ or } y), \end{aligned} \tag{3}$$

where $f_\Lambda(\mathbf{r}) = (\Lambda^2/2\pi)e^{-\Lambda^2|\mathbf{r}|^2/2}$, $1/\Lambda$ is the cut-off length scale, and $\tilde{Z}$ is the complex Fourier transform of $Z(\mathbf{r}, e = 1, B)$. As shown in Supplementary Fig. 6c, all of these quantities exhibit strong correlations with the locations of vortices. Since vortices are generally pinned at weakly superconducting regions, these results suggest that at least one of these quantities would represent the fundamental measure of the weakness of superconductivity. Although the microscopic mechanism of vortex pinning is unclear at present, the observed correlation between the vortex location and $A_{\mathrm{S}}(\mathbf{r})$ clearly indicates that the superconductivity is weak in the region where the nanostripe is prominent, indicating the competition between superconductivity and the nanostripe in $Bi_2Sr_2CaCu_2O_{8+\delta}$.

Supplementary Note 4 - Energy dependence of the intensity of the nanostripe

To identify the characteristic energy scale of the nanostripe, we study the normalized-energy $e = E/\Delta_1$ dependence of the nanostripe intensities defined as the Fourier amplitudes around $\mathbf{S}_x$ and $\mathbf{S}_y$ in Fig. 3. As shown in Supplementary Fig. 7, the nanostripe intensities gradually grow from about $e = 0.5$ and take maximum around $e = 1$, suggesting the intimate connection between the nanostripe and the pseudogap. A similar behavior has also been observed in the previous works [9,16]. It should be noted that the intensity is apparently enhanced by a magnetic field. This enhancement is more pronounced in the vortex region and quite small in the matrix region, indicating that the field enhancement is confined in the vortex core. This manifests the competition between the superconductivity and the nanostripe that is associated with the pseudogap state.

Supplementary References

[1] Hanaguri, T. *et al.* Coherence factors in a high-T_c cuprate probed by quasi-particle scattering off vortices. *Science* **323**, 923-926 (2009).

[2] Kohsaka, Y *et al.* An intrinsic bond-centered electronic glass with unidirectional domains in underdoped cuprates. *Science* **315**, 1380-1385 (2007).

[3] Hanaguri, T. *et al.* Quasiparticle interference and superconducting gap in $Ca_{2-x}Na_xCuO_2Cl_2$. *Nature Phys.* **3**, 865-871 (2007).

[4] Fujita, K. *et al.* Bogoliubov angle and visualization of particle-hole mixture in superconductors. *Phys. Rev. B* **78**, 054510 (2008).

[5] Hoffman, J. E. *et al.* Imaging quasiparticle interference in $Bi_2Sr_2CaCu_2O_{8+\delta}$. *Science* **297**, 1148-1151 (2002).

[6] Wang, Q. -H. & Lee, D. -H. Quasiparticle scattering interference in high-temperature superconductors. *Phys. Rev. B* **67**, 020511(R) (2003).

[7] McElroy, K. *et al.* Relating atomic-scale electronic phenomena to wave-like quasiparticle states in superconducting $Bi_2Sr_2CaCu_2O_{8+\delta}$. *Nature* **422**, 592-596 (2003).

[8] Kohsaka, Y. *et al.* How Cooper pairs vanish approaching the Mott insulator in $Bi_2Sr_2CaCu_2O_{8+\delta}$. *Nature* **454**, 1072-1078 (2008).

[9] Fujita, K. *et al.* Simultaneous transitions in cuprate momentum-space topology and electronic symmetry breaking. *Science* **344**, 612-616 (2014).

[10] Volovik, G. E. Superconductivity with lines of gap nodes: density of states in the vortex. *JETP Lett.* **58**, 469-473 (1993).

[11] Hayashi, N. Isoshima, T. Ichioka, M. & Machida, K. Low-lying quasiparticle excitations around a vortex core in quantum limit. *Phys. Rev. Lett.* **80**, 2921-2924 (1998).

[12] Yoshizawa, S. *et al.* High-resolution scanning tunneling spectroscopy of vortex cores in inhomogeneous electronic states of $Bi_2Sr_2CaCu_2O_x$. *J. Phys. Soc. Jpn.* **82**, 083706 (2013).

[13] Franz, M. & Tešanović, Z. Self-consistent electronic structure of a $d_{x^2-y^2}$ and a $d_{x^2-y^2}+id_{xy}$ vortex. *Phys. Rev. Lett.* **80**, 4763-4766 (1998).

[14] Kato, M. & Maki, K. Quasi-particle spectrum around a single vortex in superconductors - $d_{x^2-y^2}$-wave case -. *Prog. Theor. Phys.* **107**, 941-956 (2002).

[15] Fukuo, M., Mashima, H., Matsumoto, Y., Hitosugi, T. & Hasegawa, T. Correlation between flux pinning and inhomogeneous electronic distribution of $Bi_2Sr_2CaCu_2O_{8+\delta}$ directly probed by scanning tunneling microscopy/spectroscopy. *Phys. Rev. B* **73**, 220505(R) (2006).

[16] Lawler, M. J. *et al.* Intra-unit-cell electronic nematicity of the high-T_c copper-oxide pseudogap states. *Nature* **466**, 347-351 (2010).
